# Computed tomography features of *Stenotrophomonas maltophilia* pneumonia in patients with neutropenic fever: report of two cases

**DOI:** 10.1186/2049-6958-8-14

**Published:** 2013-02-18

**Authors:** Tsang Wai Kan Kassel, Lee Ka Lok Ryan, Allen Li

**Affiliations:** 1 Department of Radiology & Nuclear Medicine, Tuen Mun Hospital, Tuen Mun, NT, Hong Kong; 2 Department of Imaging and Interventional Radiology, Prince of Wales Hospital, Sha Tin, Hong Kong; 3 Department of Radiology, North District Hospital, Sheung Shui, Hong Kong

**Keywords:** *Stenotrophomonas maltophilia*, Stenotrophomonas, Xanthomonas, Pneumonia, Neutropenia, Neutropenic fever, Fever of unknown origin

## Abstract

*Stenotrophomonas maltophilia* (S. maltophilia) is a rare yet important global emerging nosocomial pathogen with multi-drug resistance. To the best of our knowledge, there is only one case report describing the computer tomography (CT) features of *S. maltophilia* pneumonia. In this article we will compare the features in the published case to those found in our patients. The importance of thoracic CT in febrile neutropenic patients will also be discussed.

## Background

Infection is the major cause of neutropenic fever in patients receiving chemotherapy, with the pulmonary system being most commonly involved [1]. With the advantage of being readily accessible, chest radiography (CXR) is most often the first imaging test used. However, it may not be sufficiently sensitive or specific in diagnosing pulmonary infection. Compared to CXR, thoracic computed tomography (CT) much more sensitive in the detection of pulmonary infection in immunocompromised patients [2-8]. Therefore some authors advocate the use of thoracic CT in neutropenic patients with fever of unknown origin despite initial normal CXR [9].

*Stenotrophomonas maltophilia* is an important global emerging nosocomial pathogen [10,11]. In neutropenic patients, the mortality rate of *S. maltophilia* pneumonia is up to 50%. Therefore, a prompt diagnosis of *S. maltophilia* pneumonia is essential and may improve patients' survival.

To the best of our knowledge, there is only one case report describing the CT features of *S. maltophilia* pneumonia. Here we will compare the reported findings in prior case to those found in our patients. The importance of thoracic CT in febrile neutropenic patients will also be discussed.

## Case reports

### Case 1

**Figure 1:**
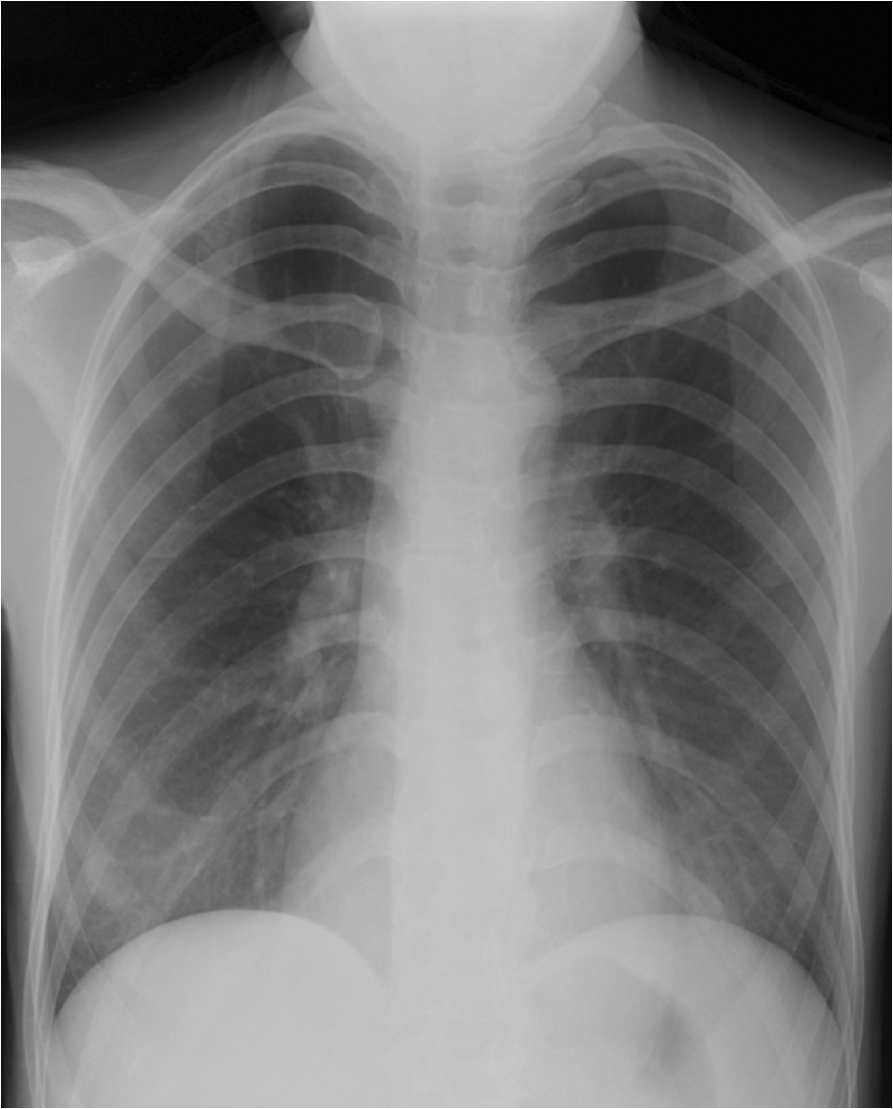
Normal chest radiograph of patient 1.

**Figure 2:**
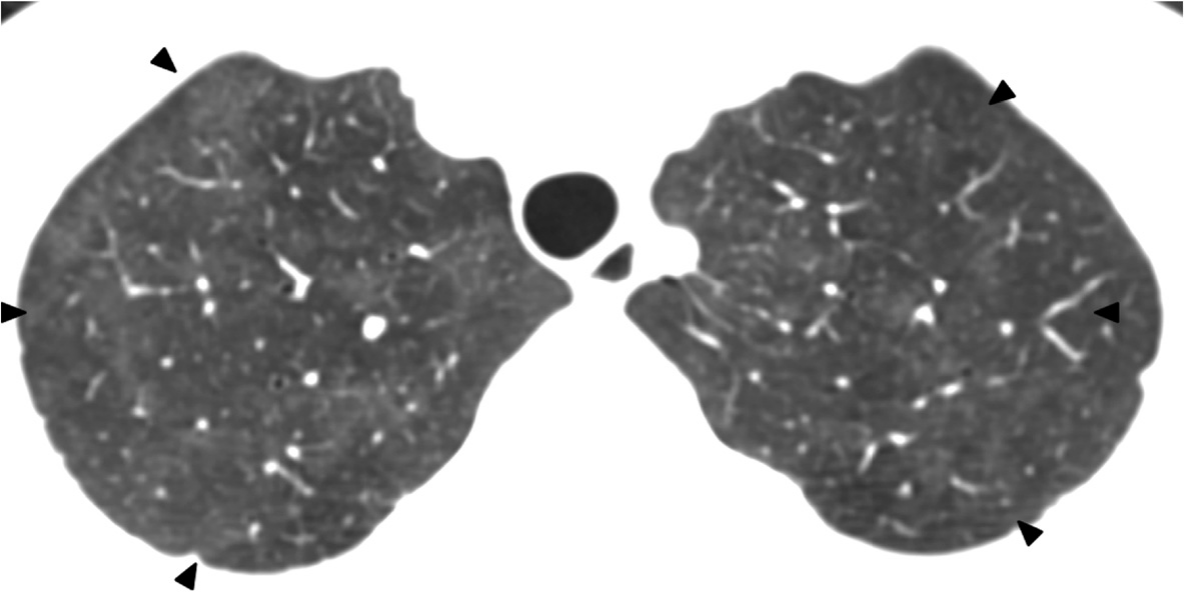
Plain thoracic CT of patient 1 shows patchy ground glass opacities at both lungs without zonal predominance.

**Figure 3:**
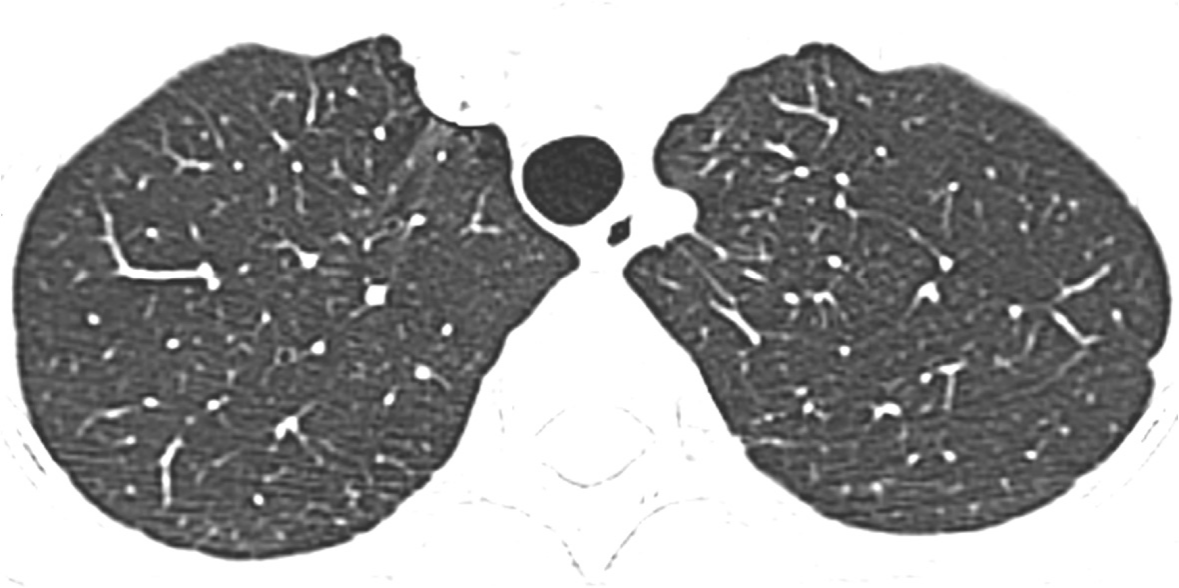
Three month follow up thoracic CT of patient 1 shows complete resolution of the ground glass opacities.

A 36-year-old lady with diffuse large B-cell lymphoma presented with neutropenic fever and cough on the 17th day of the 6th cycle of CHOP chemotherapy (cyclophosphamide, hydroxydaunorubincin, oncovin and prednisolone). Physical examination was unremarkable apart from the low grade fever (38.1°C). On admission she had a low absolute neutrophil count (ANC) (0.6x10*9/L), high erythrocyte sedimentation rate (ESR) (50 mm/h) and high C-reactive protein (CRP) level (12.3 mg/l). Chest radiograph was normal (Figure 1). Initial septic workup was negative, which included sputum bacterial culture, sputum acid fast bacilli (AFB) smear and culture, bacterial and fungal blood culture, early morning urine AFB smear and culture. Thoracic CT was performed subsequently, which showed bilateral patchy ground glass opacities without zonal predominance (Figure 2). Repeated sputum culture yielded heavy growth of *S. maltophilia*, which was sensitive to Ceftazidime, Cotrimoxazole, Levofloxacin, Ticarcillin/ Clavulanate, and resistant to Imipenem. After cessation of chemotherapy and commencement of Piperacillin/ Tazobactam (Tazocin) and Gentamycin, which later switched to Ciprofloxacin, her fever subsided and ANC normalized. Her chest radiographs were all along normal despite abnormal thoracic CT. Three months later her thoracic CT showed complete resolution of the ground glass opacities (Figure 3).

### Case 2

**Figure 4:**
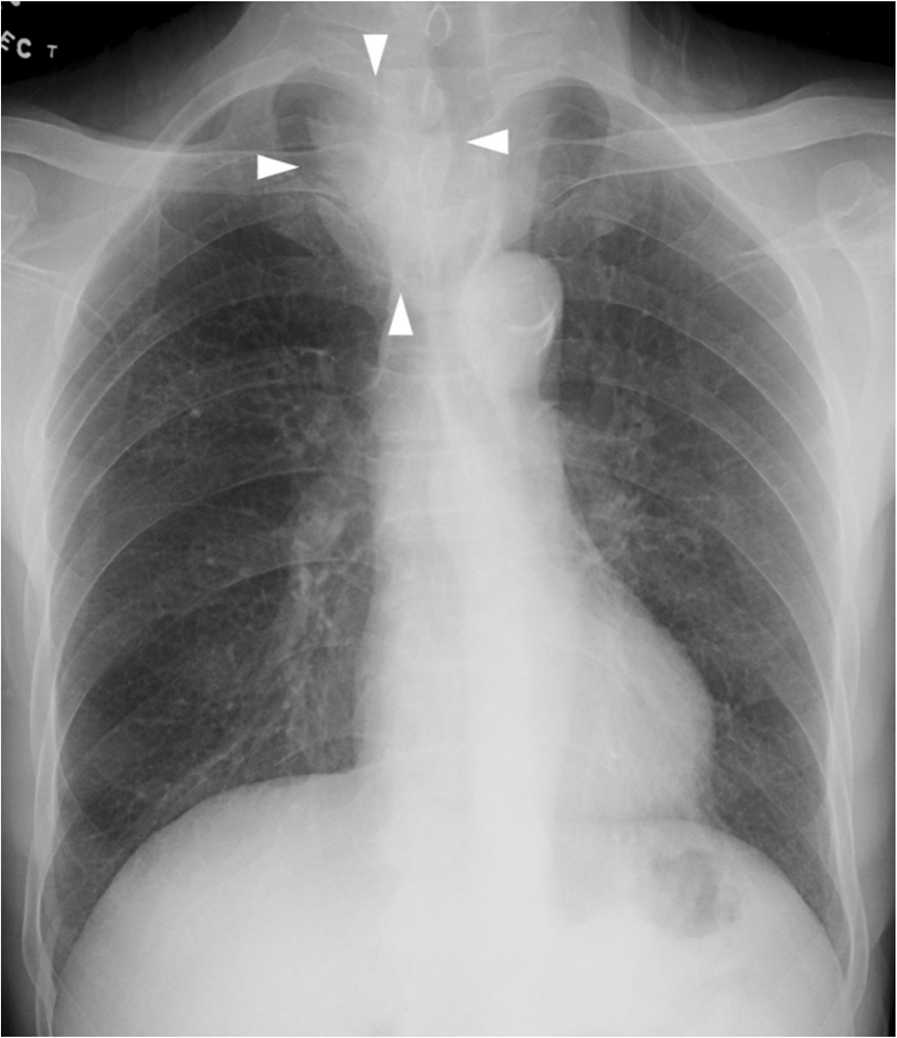
Initial chest radiograph of patient 2 shows clear lungs and right supraclavicular lymphadenopathy (arrowheads) which compresses and deviates the trachea.

A 65-year-old man with locally advanced esophageal carcinoma and right supraclavicular nodal metastasis presented with cough, dysponea and neutropenic fever on the 5th day of the 1st of palliative chemotherapy (carboplatin). His initial CXR showed clear lungs and right supraclavicular lymphadenopathy which compressed and deviated the trachea (Figure 4).

**Figure 5:**
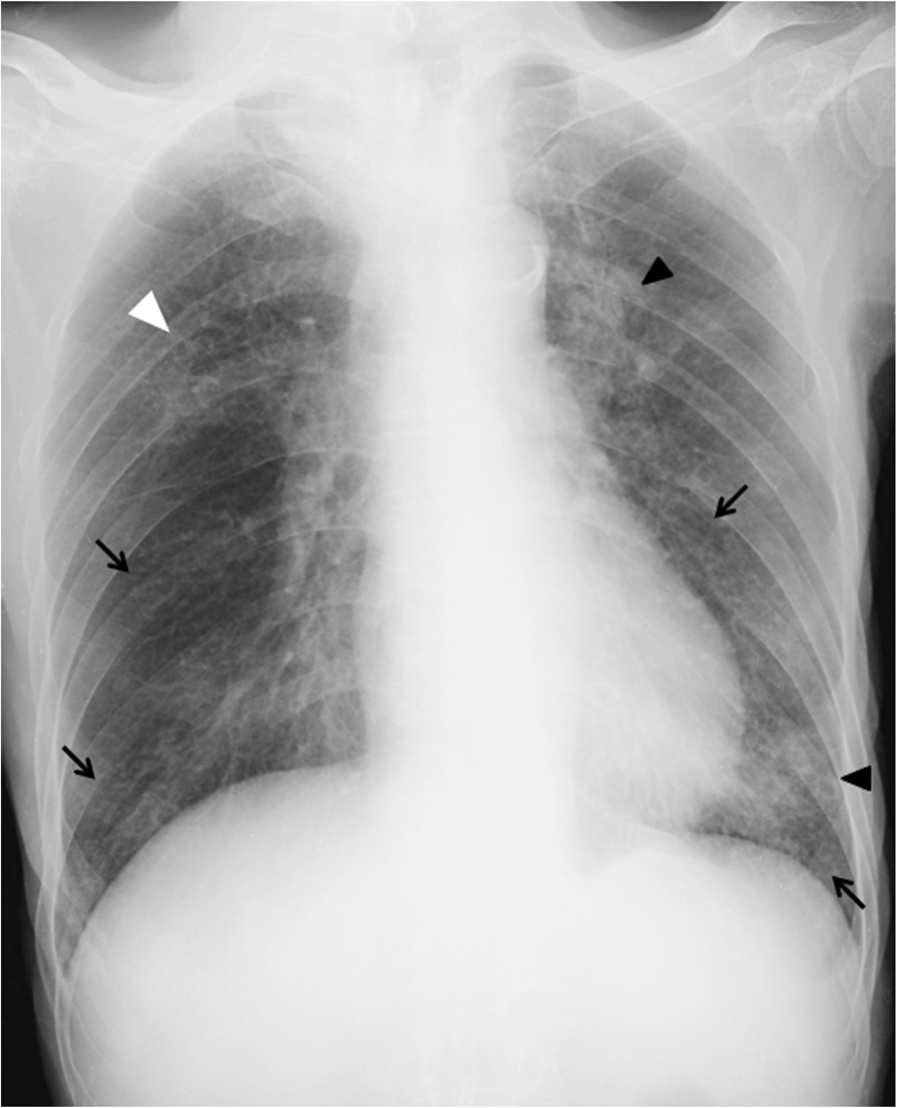
Follow up chest radiograph of patient 2 shows air space opacities (black arrowheads) at the left upper and lower zones, bronchial wall thickening (white arrowhead) at the right upper zone, and bilateral miliary nodules (black arrows).

**Figure 6:**
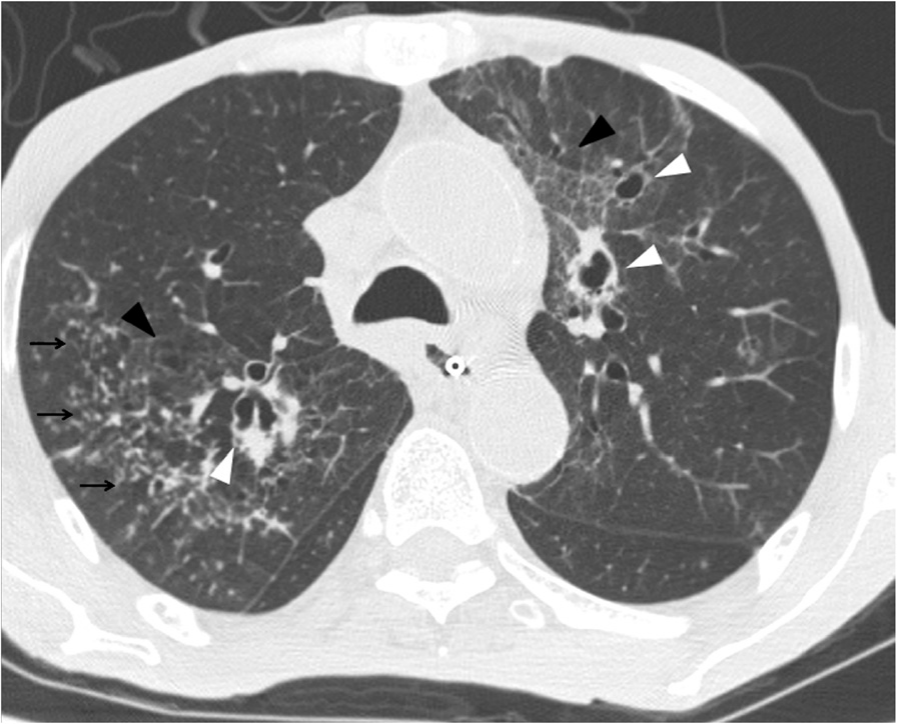
Thoracic CT of patient 2 during treatment shows bilateral ground-glass opacities (black arrowheads), numerous centrilobular nodules (black arrows), diffuse cylindrical bronchiectasis and bronchial wall thickening (white arrowheads).

**Figure 7:**
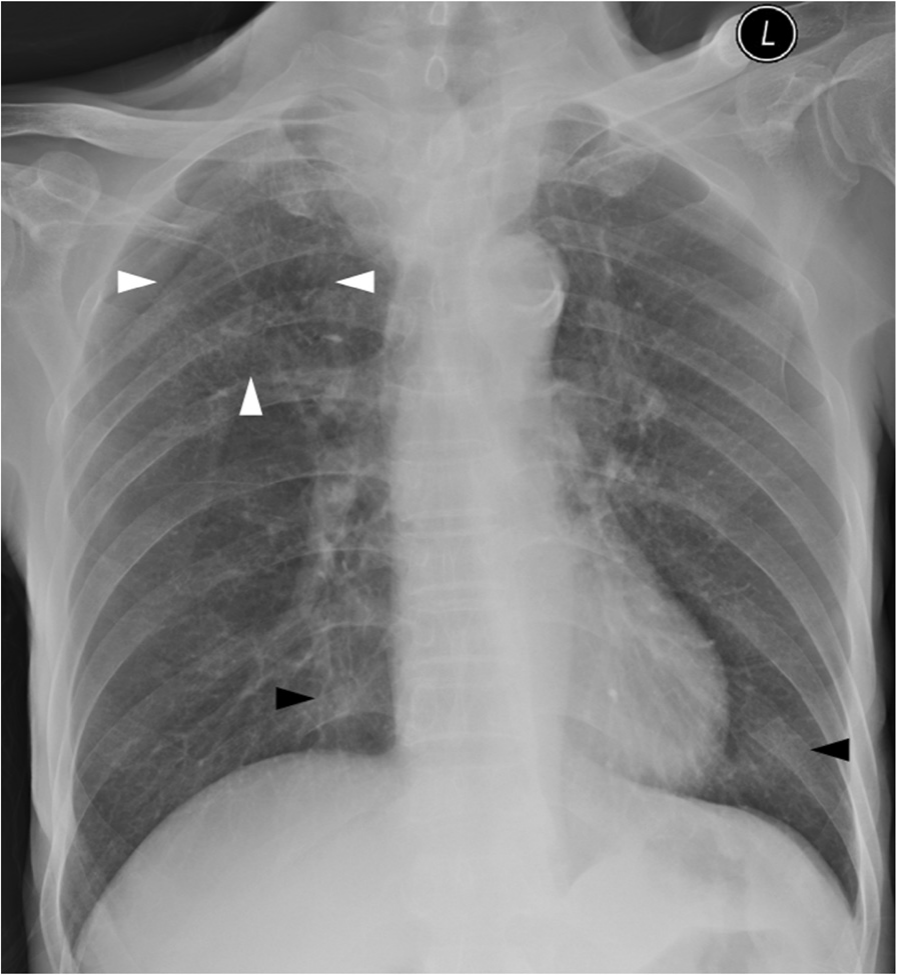
Post treatment chest radiograph of patient 2 shows only small amount of residual nodules at the right upper zone (white arrowheads) and minimal increase in lung markings at bilateral lower zones (black arrowheads).

Serial chest radiographs revealed bilateral migratory air space opacities and progressive bilateral miliary nodules (Figure 5). The initial ANC was low (0.55x10*9/L) and CRP level was elevated (66.6 mg/l). Initial septic workup was negative, which included sputum bacterial culture, sputum and bronchoalveolar lavage (BAL) AFB smear, culture, and polymerase chain reaction (PCR), nasopharyngeal aspirate viral titre and culture, blood culture, hepatitis serology, urine microscopy and culture. Thoracic CT demonstrated bilateral ground glass opacities and numerous centrilobular nodules. There was fistulation between the right supraclavicular nodal metastasis and the trachea. A diagnosis of chest infection was made. Chemotherapy was withheld and Piperacillin/ Tazobactam (Tazocin) was started as empirical treatment. Follow up thoracic CT during treatment demonstrated progression of bilateral ground-glass opacities and centrilobular nodules, and newly developed diffuse cylindrical bronchiectasis and bronchial wall thickening (Figure 6). Repeated sputum culture demonstrated heavy growth of *S. maltophilia*which was sensitive to Cotrimoxazole, Levofloxacin, Ticarcillin/ Clavulanate (Timentin), intermediate to Cefepime, Cefoperazone/ Sulbactam, Ciprofloxacin, and resistant to Ceftazidime, Imipenem, Meropenem, Piperacillin/ Tazobactam (Tazocin). The antibiotic regime was switched to Ticarcillin/ Clavulanate and Levofloxacin accordingly. The patient became afebrile and weaned off oxygen supplement after completion of a course of antibiotics. His ANA normalized and CRP level dropped. Post treatment CXR showed marked regression of the lung nodules and air space opacities (Figure 7), with only small amount of nodules remained at the right upper zone and minimal increase in lung markings at bilateral lower zone. Thoracic CT was not repeated. Despite successful treatment, the patient succumbed to advanced malignancy two months later.

Infection is the major cause of neutropenic fever in patients receiving chemotherapy, with the pulmonary system being most commonly involved [1]. Due to its high accessibility, low cost and low radiation dose, chest radiography (CXR) is most often the first imaging test used. Besides its role in detection of chest infection, CXR is also helpful in monitoring disease progress and treatment response [12-14]. According to Clinical Practice Guideline for the Use of Antimicrobial Agents in Neutropenic Patients with Cancer: 2010 Update by the Infectious Diseases Society of America, CXR is indicated in febrile neutropenic patient who is receive chemotherapy and has upper respiratory symptoms and/or cough [15]. Unfortunately, CXR might not be sufficiently sensitive or specific. It is well known that CXR can be normal even there are florid abnormalities in thoracic CT, as demonstrated in our patient 1. In most of the time, the radiographic findings are non specific and thus non-diagnostic. Compared to CXR, thoracic CT is far more sensitive in detection of pulmonary infection in immunocompromised patients [2-8]. In neutropenic febrile patients, thoracic CT can depict 20% more pneumonia and demonstrate pneumonia about 5 days earlier than CXR [9]. In other words, the diagnosis and treatment of pulmonary infection in a patient with normal CXR can be delayed till abnormalities are shown in thoracic CT. Since the prognosis of patient with neutropenic fever depends on prompt identification of causative pathogen and commencement of appropriate treatment [16,17] the role of thoracic CT in improving the prognosis of neutropenic febrile patient with pulmonary infection cannot be overstressed.

Besides being sensitive, thoracic CT is efficacious in excluding pulmonary infection. Micro-organisms are detected much more often in patients with CT features of chest infection than those with normal CT (43% versus 4%) which means that a normal thoracic CT can essentially exclude pulmonary infection [9]. In a patient with negative septic workup and persistent fever after commencement of empirical antibiotics, the cause of persistent fever can either be due to inappropriate antimicrobial regime or non-infective cause of fever, such as malignancy related fever, reaction to incompatible blood product, metabolic disorders (e.g. gout) etc. Switching antibiotic regime without direction might not be always helpful and might even cause adverse effect. Repeating full set of septic workup or carrying out invasive tests without focus may be impractical and even harmful. Thoracic CT would then be valuable at that juncture. In patient with abnormal thoracic CT, it is sensible for us to carry out detailed workup focusing on the pulmonary system. This may include some more invasive investigation with higher diagnostic yield like bronchoalveolar lavage, transbronchial biopsy of consolidative area or lymph node. In addition, some pathogens have specific CT features that allow us to reach the correct diagnosis and start appropriate treatment before positive microbiology results, for example, air crescent sign in aspergilloma, symmetrical perihilar infiltrates in Pneumocystis jiroveci pneumonia, and central necrotic lymph nodes in pulmonary tuberculosis. Since a normal thoracic CT can essentially exclude pulmonary infection, we should then put our focus on extrapulmonary causes of fever rather than launching invasive investigation on the pulmonary system. Therefore some authors advocate the use of thoracic CT in neutropenic patients with fever of unknown origin despite initial normal CXR [9].

In neutropenic febrile patients with pulmonary infiltrates, infection accounts for approximately 75% of cases. Although some pathogens have specific CT features that allow us to make the correct diagnosis, most of them do not and the final diagnosis of the offending organism still relies on the microbiology results.

*Stenotrophomonas (Xanthomonas) maltophilia* is an aerobic, gram-negative bacillus with multi-drug resistance which is closely related to the Pseudomonas species. Being an opportunistic pathogen in immunocompromised patients, it is emerging globally in recent years [10]. It can cause serious infections in various parts of the body, with the respiratory tract (33%) and central catheter (31%) being most commonly involved [10,11]. There is a significantly higher risk of developing *S. maltophilia* disseminated infection in patients having advanced immunodeficiency, neutropenia, central venous catheterization, prior broad-spectrum antimicrobial therapy and/or corticosteroid treatment, intubation or tracheotomy, prolonged hospitalization and severe mucous lesions [10,18]. Concerning our patients, both of them had the risk factors of being immunocompromised and neutropenic due to intrinsic malignancy and chemotherapy treatment. The overall mortality rate of neutropenic patient with *S. maltophilia* pneumonia is up to 50%, which is further increased to >80% in those requiring endobronchial intubation [19]. Therefore a prompt diagnosis is essential in treatment planning and may improve patient’s morbidity and mortality.

Apart from infection, there are other differential diagnoses for pulmonary infiltrates in patients with neutropenic fever. These include radiation pneumonitis, drug toxicity and diffuse alveolar hemorrhage. All of them can show similar radiological findings as those in pneumonia. Radiation pneumonitis should be considered if there is a history of radiation therapy to the chest. In general it develops within 4–12 weeks after completion of radiation therapy in patients receiving more than 40 Gy of radiation. In the acute phase it presents as ground-glass opacities or consolidation. In the late phase traction bronchiectasis, volume loss, and consolidation can be found [20]. Consolidation with geographic straight border that confines within the radiation port is characteristic. In pulmonary drug toxicity, there is bilateral hetero- or homogeneous ground glass opacities, usually in the mid and lower lungs [21]. Diffuse alveolar haemorrhage (DAH) is an uncommon complication caused by certain medications, typically anticoagulants and cytotoxic drugs likely high-dose cyclophosphamide and cytarabine [22]. It has a high mortality rate, thus prompt diagnosis is important. Imaging features consist of bilateral scattered or diffuse areas of ground-glass opacity or consolidation. In most cases these patients present with acute respiratory distress [21]. A drop of haematocrit level is a hint to the correct diagnosis, and the presence of progressive hemorrhagic bronchoalveolar lavage is diagnostic.

To the best of our knowledge, there is only one case report describing the CT features of *S. maltophilia*[23]. In that case, multifocal areas of air-space consolidation, ground-glass attenuation areas, small centrilobular nodules and bronchial wall thickening were present. In our series, the sole CT finding of patient 1 is bilateral patchy ground glass opacities without zonal predominance. Air-space consolidation, ground glass opacities, numerous centrilobular nodules and bronchial wall thickening are noted in patient 2. These are similar to those described in prior case report. Compared with that reported case, the only new imaging finding in patient 2 is bilateral cylindrical bronchiectasis. Among all three cases, diffuse ground glass opacities without zonal predominance is the most consistent CT features of *S. maltophilia* pneumonia. Other imaging findings such as bilateral multifocal areas of air-space consolidation and centrilobular nodules can be occasionally found. Bronchiectasis likely represents the later stage of pneumonia. However, since these imaging features and pattern are not specific nor diagnostic, the final diagnosis of *S. maltophilia* pneumonia still relies on microbiology results.

## Conclusions

*Stenotrophomonas maltophilia* is a rare yet important globally emerging opportunistic pathogen. The most consistent CT features of *S. maltophilia* pneumonia is diffuse ground glass opacities without zonal predominance. Bilateral multifocal areas of air-space consolidation, centrilobular nodules, bronchiectasis and bronchial wall thickening are occasionally found. Due to its supreme sensitivity, the role of thoracic CT in the detection and exclusion of pulmonary infection, including *S. maltophilia* pneumonia, in neutropenic febrile patients cannot be overstressed.

## Consent

Written informed consent was obtained from the patient for publication of this report and any accompanying images.

## Competing interest

The authors declare that they have no competing interests.
